# Real-World Performance of COVID-19 Antigen Tests: Predictive Modeling and Laboratory-Based Validation

**DOI:** 10.2196/68376

**Published:** 2025-10-06

**Authors:** Miguel Bosch, Dawlyn Garcia, Lindsey Rudtner, Nol Salcedo, Raul Colmenares, Sina Hoche, Jose Arocha, Daniella Hall, Adriana Moreno, Irene Bosch

**Affiliations:** 1 Info Analytics Innovations, Houston, TX, United States; 2 IDX20 Inc, 166 Clinton Rd, Brookline, MA, 02445, United States, 1 6177347745

**Keywords:** COVID-19, SARS-CoV-2 antigen test, lateral flow assay, point-of-care diagnostics, real-world performance, Langmuir–Freundlich isotherm, Bayesian regression (Monte Carlo), probability of positive agreement, limit of detection, image-based signal quantification

## Abstract

**Background:**

Rapid and safe deployment of lateral-flow antigen tests, coupled with uncompromised quality assurance, is critical for outbreak control and pandemic preparedness, yet real-world performance assessment still lacks laboratory and quantitative approaches that remain uncommon in current regulatory science. The approach proposed here can help standardize and accelerate early phase appraisal of antigen tests in preparation for clinical validation.

**Objective:**

The aim of this study is to present a quantitative, laboratory-anchored framework that links image-based test line intensities and the population distribution of naked-eye limits of detection (LoD) to a probabilistic prediction of positive percent agreement (PPA) as a function of viral-load–related variables (eg, quantitative real-time polymerase chain reaction [qRT-PCR] cycle thresholds [Cts]). Using dilution-series calibrations and a Bayesian model, the predicted PPA-vs-Ct curve closely tracks the observed PPA in a real-world self-testing cohort.

**Methods:**

The proposed methodology combines: (1) a quantitative evaluation of the test signal response to concentrations of target protein and inactive virus or active virus, (2) a statistical characterization of the LoD using the observer’s visual acuity of the test band, and (3) a calibration of a gold-standard method (eg, qRT-PCR cycles) against virus concentration. We elaborate these quantitative methods and unfold a Bayesian-based predictive model to describe the real-world performance of the antigen test, quantified by the probability of positive agreement as a function of viral-load variables like qRT-PCR Cts.

**Results:**

We applied the methodology by characterizing each brand of COVID-19 antigen test and estimating its real-world probability of agreement with qRT-PCR. We aligned protein and inactivated-virus standard curves at matched signal intensities and fit a linear calibration linking protein to viral concentrations. Using logistic regression, we modeled the PPA as a continuous function of qRT-PCR Ct, then integrated this curve over a predefined reference Ct distribution to obtain the expected sensitivity. This standardization enables consistent performance comparisons across sites.

**Conclusions:**

Modeling performance under real-world conditions requires coupling laboratory evaluation with the population’s ability to perceive the test’s visual signal. We represent observer capability as a probability density function of the LoD over the signal-intensity domain. Rather than reporting bin-based sensitivity, we summarize performance with the PPA as a continuous function of qRT-PCR Ct. Our framework produces PPA–Ct curves by composing (1) normalized signal-to-concentration models from the laboratory, (2) the observer LoD distribution, and (3) a Ct-to-viral-load calibration. The resulting inferences are inherently context-bound—disease-, assay-, and setup-specific. External validity depends on the particular antigen lateral-flow test, the user population (visual acuity and interpretation), and cross-laboratory qRT-PCR calibration. Comprehensive clinical studies under intended-use conditions are still required before making generalized claims.

## Introduction

Quantifying the performance of antigen lateral flow tests (Ag-LFT) according to the US regulatory science standards commonly requires the calculation of test performance statistics—for example, sensitivity or positive percent agreement (PPA) of an antigen test’s (AT) binary assessments with reference to the quantitative real-time polymerase chain reaction (qRT-PCR) gold standard [1-3]. These statistics are based on human clinical samples, requiring paired qRT-PCR cycle thresholds (Cts) to generate performance data and corresponding test validations as described in a comprehensive literature review for COVID-19 studies [4]. The clinical performance of Ag-LFTs increases at higher viral loads (low Ct values), which present early on in the symptom window, and test performance declines with low viral loads (high Ct values) at the end of the acute disease window [2,5-10]. These realities motivate a fast, laboratory-anchored, model-based appraisal that anticipates PPA-vs-Ct before large trials. That is, AT performance is closely related to the sample viral load and the performance statistics are dependent on several factors such as viral load distribution, specific virus variants [8,9,11], and symptomatic versus asymptomatic cases [7,12], as well as the observer’s training [6,13-17]. Hence, the regulatory process is typically a lengthy process and includes appropriate sample size and requires a viral load distribution to cover the spectrum of target concentrations.

Because Ag-LFTs are powerful tools for transmission control and epidemic mitigation, the fast and safe deployment of tests without compromising quality assurance in the evaluation process is key for outbreak control and pandemic preparedness. Home testing and easy access to Ag-LFTs enables them to be used for serial testing, as has been recommended. For COVID-19 Ag-LFTs, serial testing increases effective sensitivity [18], including in home testing [7,13,19].

We have developed a methodology for the quantitative evaluation of SARS-CoV-2 ATs based on laboratory measurements of the regions of interest (ROIs), including the test’s regions and corresponding normalized signal intensity and binary naked-eye user assessments for positive or negative results. In both cases, we characterized the test performance according to the sample concentration of the target recombinant protein, with heat-inactivated virus as well as biologically active virus, and we used human samples self-collected 2 times per week, under a prospective clinical protocol (ClinicalTrials.gov: NCT05884515). To support accurate self-reporting, participants received a brief, standardized orientation with visual aids on test interpretation, photo capture, and upload procedures. Including the statistical characterization of the user population’s limit of detection (LoD) in the signal intensity domain, we developed a predictive model for the probability of positive agreement in real-world conditions.

Our method involves (1) characterizing the AT signal intensity with protein and inactivated virus dilutions, (2) calibrating the qRT-PCR cycles with virus dilutions, (3) characterizing the signal intensity LoD of the user population for the AT, and (4) predicting the real-world probability of a positive agreement signal response of the AT. Our methods have the advantage of being formulated using continuous variable analysis and probability models instead of plain discrete analysis and sample statistics.

We demonstrate our methodology capabilities when comparing the predicted probability of positive agreement with that generated using real-world data collected through an institutional review board (IRB)–approved study for frequent antigen testing to monitor COVID-19 in an underserved population (ClinicalTrials.gov: NCT05884515). Participants consisted of individuals from vulnerable populations in low-income and assisted-living facilities located in the city of Chelsea, MA. Recruitment included people living in state-regulated, independent senior living communities and other residents of Chelsea. The consented participants were provided with ATs to routinely self-test for COVID-19 at home or in community centers, 2 times per week, uploading the test results and photos to the project informatics platform. We obtained confirmatory qRT-PCR data for all positive results detected by the home AT and for a random number of negative results from an independent Clinical Laboratory Improvement Amendments laboratory. The certified Clinical Laboratory Improvement Amendments laboratory procedures were approved by the IRB and the qRT-PCR data shared included the Ct values for each submitted test.

We describe the quantitative analysis of the AT for signal intensity and naked-eye binary data, the characterization of the user’s LoD, the calibration of the qRT-PCR, and the formulation of the predictive model. In the Results section, we illustrate the application of the described methodology with the characterization of an AT in the common cassette device presentation and compare the predicted result with the real-world probability of positive agreement.

## Methods

### Overview

**Figure 1.**
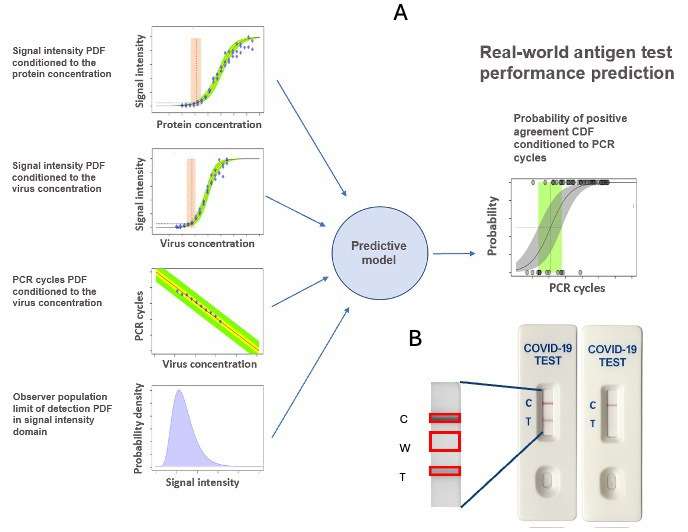
(A) Schematic of the inference model. Input components are based on various laboratory calibrations of the antigen test–normalized signal intensity against protein and/or virus concentrations, qRT-PCR Cts against virus concentrations, and the limit of detection PDF of the observer population’s visual assessment of the test result. The model predicts performance for real-world conditions of the antigen tests, quantified as the probability of agreement function. (B) Schematic of the antigen test results as positive or negative (recombinant antigen and/or inactivated virus or patient sample), according to the manufacturer’s protocol and read at the specified development time. The cassette image was generated using a cell phone camera, and the annotated regions of interest were the control band (C), local white background (W), and test band (T) areas. Mean gray scale values from these regions of interest are used to compute normalized signal intensity for model fitting. Ct: cycle threshold; PDF: probability density function; PPA: positive percent agreement; qRT-PCR: quantitative real-time polymerase chain reaction.

The present methodology combines (1) a quantitative evaluation of the test signal response to concentrations of target protein and inactive or active virus, (2) a statistical characterization of the LoD using observers’ visual acuity of the test band, and (3) a calibration of a gold-standard method (ie, qRT-PCR cycles) against virus concentration. We elaborate these quantitative methods and unfold a Bayesian-based predictive model to describe the real-world performance of the AT, quantified by the probability of positive agreement as a function of viral-load variables like qRT-PCR Cts. Figure 1A describes the different types of information involved in the predictive model.

### AT Signal Response Characterization

In a selected lateral flow test, we measured the signal intensity of the test, white background, and control band, captured digitally in a photograph using a cell phone camera (Figure 1B). We processed this image and evaluated the average pixel intensity in 3 ROIs on the nitrocellulose strip. The resulting gray scale–normalized signal intensity is a continuous variable independent of the observer. There are existing methodologies to assess AT performance, such as test band signal intensity [20] and LoD studies [21]. We calculated the signal intensity by subtracting the white background and test band average pixel brightness and normalizing by the largest signal intensity present in the dataset. The software was designed to provide use instructions, obtain gray scale pixel intensity of the ROI, compute normalized pixel intensities, and generate a written report [22].

We analyzed the signal intensity response of an AT, calculating the signal intensity across a dilution series of the target recombinant protein. To fit these data, we used techniques based on isotherm modeling [23,24] and used the Langmuir-Freundlich adsorption model [25,26],

 , (1) $$I=\frac{k\;C^{b}}{1+k\;C^{b}}$$

with I being the normalized signal intensity, C the concentration, k the adsorption equilibrium constant, and b an empirical exponent close to one. The model parameters to estimate by fitting the normalized intensity data corresponded to the adsorption constant k and the exponent b. We used a Bayesian regression solved with Monte Carlo sampling, which provided a description of the model uncertainties. We followed a similar procedure to characterize the relationship of the normalized signal intensity with other variables in addition to that of using recombinant protein concentrations (eg, with a known plaque-forming unit/mL of SARS-CoV-2 followed by chemical inactivation of the virus).

### Probability of Agreement Function in Naked-Eye Assessment

A common use of ATs involves human naked-eye interpretation of the result. The outcome of each assessment is a binary variable, either positive or negative (1 for positive or 0 for negative for mathematical analysis). We considered that for the naked-eye analysis, individuals required training to follow specific protocols to properly report test results. Before self-testing, participants completed a brief, standardized orientation (10‐15 min) delivered in English or Spanish according to specifications of the Chelsea study. The module covered (1) correct nasal specimen collection and adherence to the manufacturer’s instructions for use, (2) strict timing of the development/read window, (3) interpretation of positive, negative, and invalid outcomes, and (4) photographing and uploading results from a cell phone camera or tablet to the study’s digital platform in real time. Participants received a 1-page pictorial quick-start guide. In addition, refresher prompts were available on the study platform. Thereby, efforts were made to stabilize the observer LoD distribution data used in our model.

The PPA or sensitivity of a test is a well-known measure of test performance. The PPA is strongly dependent on the viral concentration distribution of the tested samples (eg, sensitivity improves with higher concentrations of the target).

For an accurate description of the naked-eye performance of the test, we estimated the PPA as a *function* of the nucleoprotein concentration or other viral concentration–related variable, such as qRT-PCR Cts [8]. We modeled the PPA with a logistic function,

, (2) $$p\left(x\right)=\frac{1}{1+e^{-\left(a+bx\right)}}$$

with x being the viral-load–related variable and p(x) being the probability of positive agreement function. The model parameters to estimate fitting the binary naked-eye data are the intercept a and the slope b. The PPA function is commonly described against qRT-PCR cycles; similarly, we applied this method for other viral-load–related variables (like concentration and normalized signal intensity).

### Predictive Model for the Probability of Positive Agreement

The formulation of the model followed a probabilistic approach, meaning that variables and relationships across the model were randomized and described by probability density functions (PDFs). We defined random variables used in this formulation. There was a group of continuous positive variables that were related to the viral load: the recombinant protein concentration $x_{prot}$, the virus concentration $x_{vir}$, the test–normalized signal intensity $x_{int}$, the observer LoD–normalized signal intensity $x_{lod}$, and the qRT-PCR cycles $x_{cycle}$. In addition, we had the binary agreement variable *A,* which indicated the observer assessment of the test outcome, with values of 0 (for negative) or 1 (for positive).

For the purpose of this analysis, the LoD did not represent an exact value. We analyzed the LoD associated with a group of observers (ie, a certain population) or a single observer; LoD depends on different environmental circumstances (eg, illumination, visual context) and individual abilities. Hence, we consider the LoD as a random variable defined by a PDF $p_{LoD}(x_{lod})$ in the domain of the normalized signal intensity $x_{int}$. The probability of positive agreement (ie, the conditional positive agreement PDF) was the corresponding cumulative distribution function of the LoD PDF,

. (3) $$p\left(A=1|\;x_{int}\right)=\;\int_{0}^{x_{int}}\;p_{LoD}\left(x_{lod}\right)\;\;dx_{lod}$$

Correspondingly, the LoD PDF in the signal intensity domain was the derivative of the probability of positive agreement in the same domain. It summarized the process of observation and assessment of the testing device by the observer or the observer population.

### Probability of Positive Agreement Across Viral-Load–Related Domains

**Figure 2.**
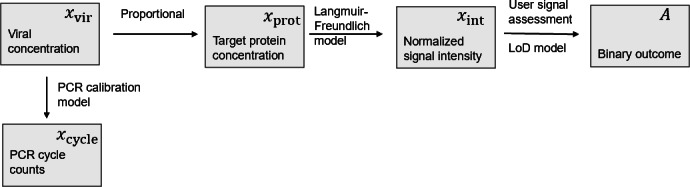
Bayesian network showing (boxes) the model random variables, (arrows) their causal relations and (annotations) relation models. LoD: limit of detection; PCR: polymerase chain reaction.

To follow, we transformed the probability of agreement in the signal intensity domain (expression 3) to the rest of the viral-load–related domains: recombinant protein concentration, viral concentration, and qRT-PCR cycles. The defined random variables and their causal dependencies are described in the Bayesian network of Figure 2. Let us consider, first, the case of the protein concentration domain. As per the intensity analysis described previously, we experimentally estimated a conditional probability for the signal intensity given the recombinant protein concentration $p\left(x_{int}|x_{prot}\right)$. We used this information to propagate the probability of positive agreement from the signal intensity domain to the protein concentration domain. For this purpose, we applied the probability chain rule,

. (4) $$p\left(A,\;x_{\text{int}}|x_{\text{prot}}\right)=p\left(A|x_{\text{int}},\;x_{\text{prot}}\right)\;p\left(x_{\text{int}}|x_{\text{prot}}\right)$$

Integrating in the $x_{int}$ domain and taking into account that the observer assessment is only dependent on normalized signal intensity (see Figure 2) $p\left(A\;|\;\;x_{int}\;,\;\;x_{prot}\right)=p\left(A\;|\;x_{int}\right)$,

. (5) $$p(A|x_{\text{prot}})=\int p(A|x_{\text{int}})\;p(x_{\text{int}}|x_{\text{prot}})\;dx_{\text{int}}$$

The functions within the integral involve the PPA in the signal intensity domain (expression 3) and the PDF of signal intensity conditioned to the protein concentration. We determined the integral by Monte Carlo integration. Similarly, we transformed the probability of agreement to the virus concentration domain and qRT-PCR cycles domain as


(6) $$p(A|x_{\text{vir}})=\int p(A|x_{\text{int}})\;p(x_{\text{int}}|x_{\text{prot}})\;p(x_{\text{prot}}|x_{\text{vir}})\;dx_{\text{int}}\;dx_{\text{prot}}$$

and

. (7) $$p(A|x_{\text{cycle}})=\int p(A|x_{\text{int}})\;p(x_{\text{int}}|x_{\text{prot}})\;p(x_{\text{prot}}|x_{\text{vir}})\;p(x_{\text{vir}}|x_{\text{cycle}})\;dx_{\text{int}}\;dx_{\text{prot}}\;dx_{\text{vir}}$$

To model the probability of agreement in the domain of qRT-PCR cycles, as is common for real-world testing, we solved Equation 7 by Monte Carlo integration. For this purpose, we needed to estimate models for the 4 conditional probabilities within the right-hand integrand, which involve the information represented in Figures1  2. In our implementation, we first integrated in the virus concentration domain to have a relationship between the protein concentration and the qRT-PCR cycles, $p\left(x_{prot}\;|\;x_{cycle}\right)$. Thus,

. (8) $$p(A|x_{\text{cycle}})=\int p(A|x_{\text{int}})\;p(x_{\text{int}}|x_{\text{prot}})\;p(x_{\text{prot}}|x_{\text{cycle}})\;dx_{\text{int}}\;dx_{\text{prot}}$$

The conditional probability of positive agreement $p\left({A|x}_{cycle}\right)$ fully describes the test performance in the qRT-PCR cycle domain. For a given PDF of the sample polymerase chain reaction (PCR) cycles distribution, the resulting sensitivity $p\left(A\right)$ is by integration,

. (9) $$p(A=1)=\int p(A=1|x_{\text{cycle}})\;p(x_{\text{cycle}})\;dx_{\text{cycle}}$$

For a given collection of N real-world samples with PCR cycles $x_{cycle}=\left\{x_{1\;},\;x_{2\;},\ldots x_{n\;},\ldots ,\;x_{N\;}\right\}$ , the previous integral is approximated by the average of the PPA function evaluated at the sample qRT-PCR cycles,

. (10) $$p\left(A=1\right)\;\approx \;\frac{1}{N}\;\sum_{1}^{N}p\left(A=1\;|\;x_{n}\right)$$

We have made software available to calculate the probability of agreement [22].

### Ethical Considerations

This study was reviewed and approved by Advarra IRB for the protocol “Center of Complex Interventions – IDx20-001, Community frequent antigen testing to monitor COVID-19 in senior public housing setup (Pro00059157).” The most recent continuing review approval was granted on November 13, 2023, with an approval period through November 13, 2024. Advarra attests compliance with the US Department of Health and Human Services 45 CFR 46 and Food and Drug Administration 21 CFR 50/56 and is registered with OHRP/FDA (IRB number 00000971). All procedures adhered to the ethical standards of the responsible institutional/national committees and the World Medical Association Declaration of Helsinki. All personnel received certification and training through the Collaborative Institutional Training Initiative for human subjects research protection. There was no compensation to participants.

All study personnel completed Collaborative Institutional Training Initiative coursework prior to engaging in any human-subjects activities. This training is required by our IRB and institutional policy and included role-appropriate modules in Biomedical Human Subjects Research, Good Clinical Practice, Responsible Conduct of Research, Conflicts of Interest, and Health Insurance Portability and Accountability Act Privacy/Security. Certificates were verified by the private investigator and maintained on file. The curriculum covers the Belmont Report principles and applicable regulations (45 CFR 46 and Food and Drug Administration 21 CFR Parts 50/56), informed consent and documentation, recruitment and equitable selection, protection of vulnerable populations, adverse event and deviation reporting, data privacy/confidentiality, and secure data management.

Because the protocol involves point-of-care antigen testing and handling of respiratory specimens, staff also completed biosafety/Blood borne Pathogen training and followed BSL-2-appropriate standard operating procedures. All participant-facing procedures (screening, consent, anterior-nares swab collection, test execution, results disclosure) were conducted only by trained personnel under IRB-approved standard operating procedures. Study data were coded with limited identifiers, stored in access-controlled databases, and managed according to least-privilege access and audit-trail requirements. This statement documents personnel competence and compliance with human-subjects protections for the conduct of this study.

Prior to study procedures, all participants were informed of the study purpose, procedures, potential risks and benefits, data uses, and their right to withdraw without penalty. Written informed consent was obtained from each participant using IRB-approved consent materials. No identifiable personal information is reported in this manuscript.

Data were collected and stored using IRB-approved procedures designed to protect participant privacy and confidentiality; only deidentified or aggregated data are presented. For studies of internet/digital tools, we complied with applicable local, national, and international regulations on the protection of personal information, privacy, and human rights.

Any protocol amendments, consent-form changes, or substantive reportable events (eg, unanticipated problems, adverse device effects, or protocol violations affecting rights, safety, or data integrity) were submitted to Advarra in accordance with IRB requirements prior to implementation.

## Results

We illustrate the application of the described methodology to characterize the analyzed COVID-19 AT brand and predict the corresponding real-world probability of agreement against qRT-PCR data.

**Figure 3.**
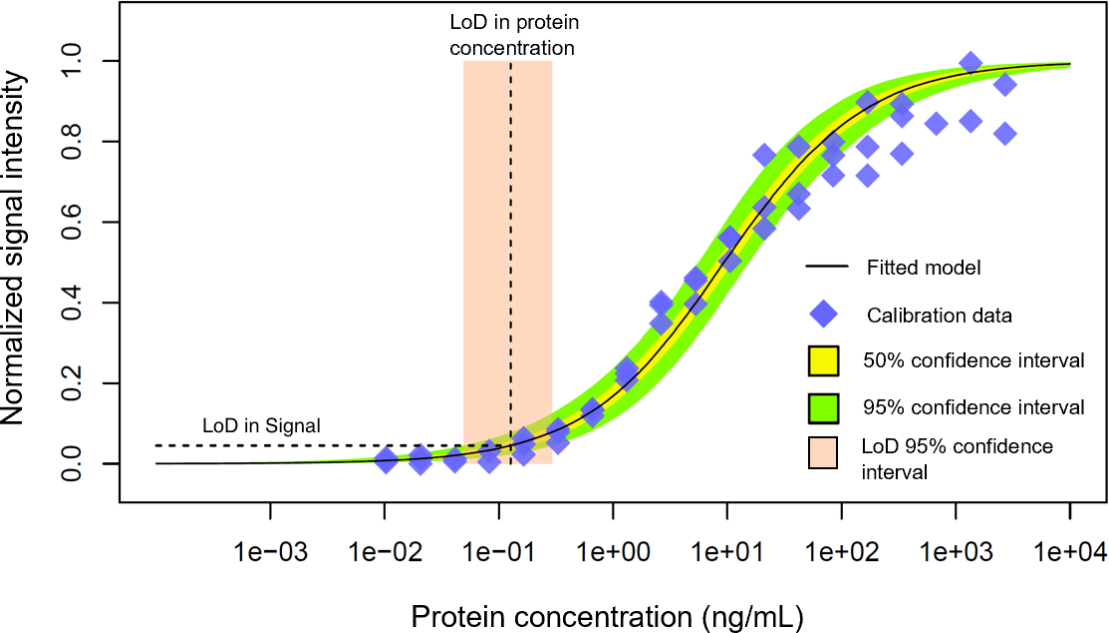
Normalized signal intensity data for 3 series of protein dilution curves for one of the COVID-19 test brands and Langmuir-Freundlich model. The estimation of the LoD (LoD confidence intervals at 95%). LoD in signal intensity was 5% in the normalized signal intensity. LoD: limit of detection.

**Figure 4.**
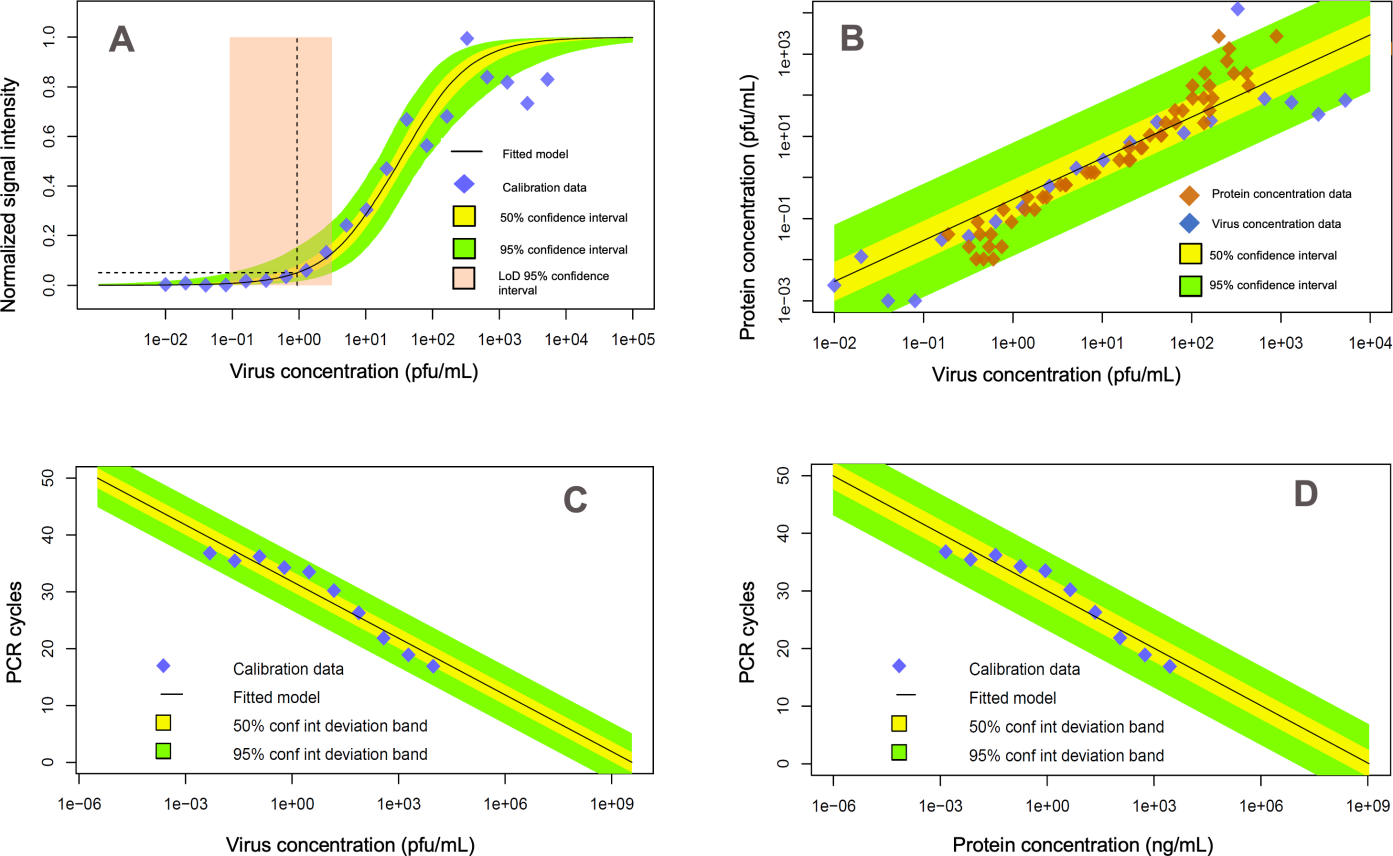
Characterization of the signal response with inactivated virus and domain-related transformation functions calibrated from experimental data. (A) The normalized signal response analysis for serial dilutions of inactivated virus. (B) Protein and virus concentration linear relational model based on the common signal intensity response of the devices, which allows transforming virus concentration to protein concentration and vice versa. (C) PCR cycle response calibration to inactivated virus dilution series. (D) qRT-PCR cycle response related to protein concentration, by combining transformations (B) and (C). LoD: limit of detection; PCR: polymerase chain reaction; pfu: plaque-forming unit; qRT-PCR: quantitative real-time polymerase chain reaction.

Figure 3 shows the signal intensity data corresponding to protein dilutions prepared in the laboratory for this AT and the corresponding Langmuir-Freundlich regression model; from the analysis, we modeled the conditional PDF $p\left({x_{int}|x}_{prot}\right)$. Figure 4 illustrates our modeled relationships across various viral-load–related variables based on our experimental characterization of the AT and qRT-PCR calibration. Figure 4A shows the signal intensity analysis of the AT based on serial dilutions of inactivated virus. The plot is similar to Figure 3, which shows the signal response to protein dilutions. By combining the protein and virus curves for common signal intensity responses, we calibrated a linear model that describes the relationship between protein and inactivated virus concentration (Figure 4B). Figure 4C shows the calibration of the qRT-PCR Ct curve based on PCR-analyzed inactivated virus dilution series. The qRT-PCR analysis of the dilution series was conducted by the same center used for self-testing our ordinary (real-world) testing program. Figure 4D shows the relationship between qRT-PCR cycles and protein concentration, transformed from the viral concentration domain by the protein-virus relationship characterized in Figure 4B. All the relationships shown in Figure 4 are modeled as conditional PDFs; for illustration, the plots show specific confidence limits.

**Figure 5.**
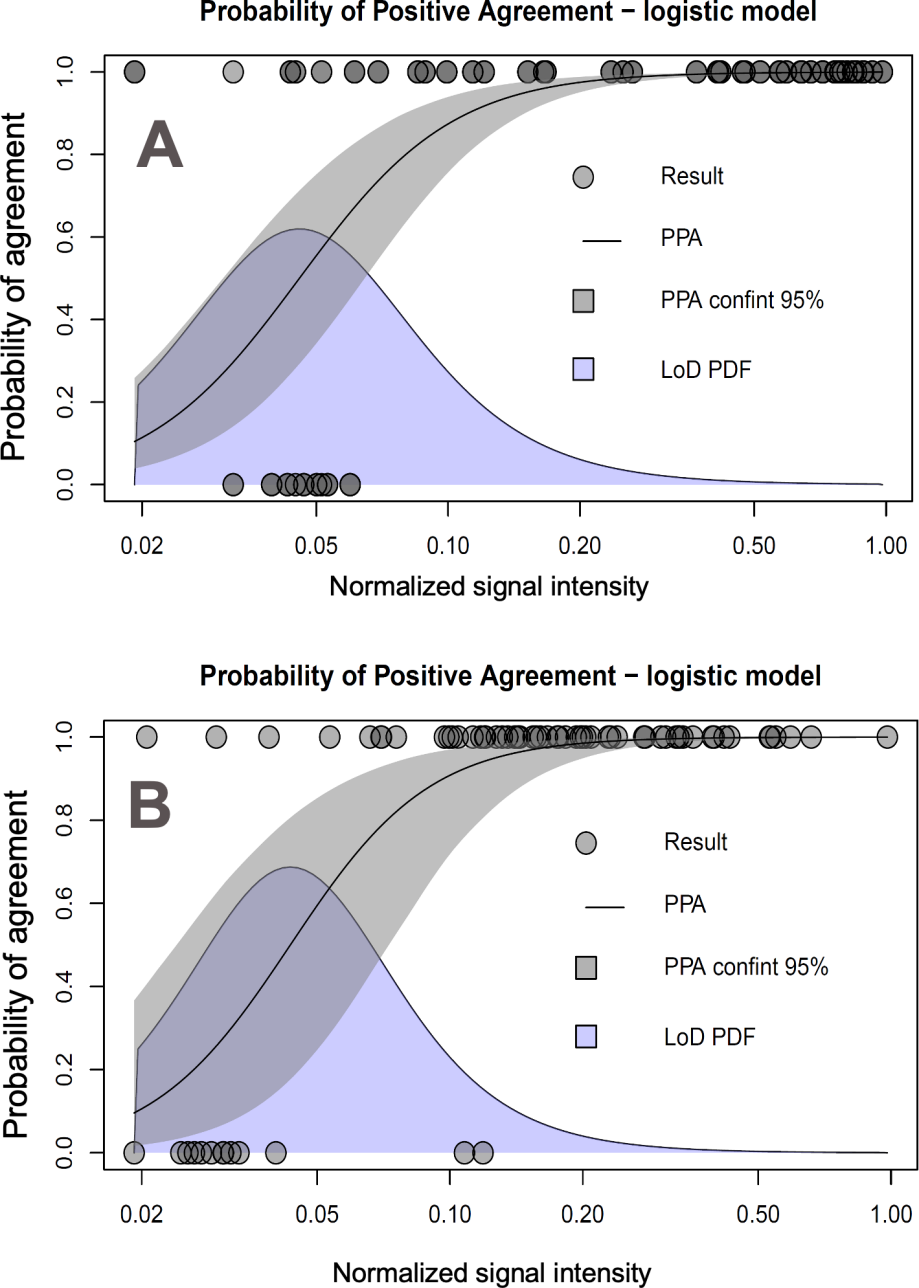
Probability of positive agreement and LoD probability density for naked-eye assessments of antigen tests in common cassette presentation made by two different groups of observers. (A) Trained staff observing results from SARS-CoV-2 nucleoprotein dilutions blinded to the observer and (B) community participants of the study reporting self-tested results. In the case of trained staff, normalized intensity was calculated from laboratory environment photographs using cell phone cameras. The community participants’ mobile phone photographs were uploaded to the digital reporting system and the visual assessment was a self-report also recorded in real time through the study’s digital platform. LoD: limit of detection; PDF: probability density function.

An additional component required by our predictive method is the LoD PDF of the observer’s population $p_{LoD}(x_{lod})$ in the domain of the signal intensity. Figure 5A shows the real-world binary assessment conditioned to the signal intensity (based on Chelsea study participants’ AT results and uploaded cell phone camera photos), the corresponding PPA function of the signal intensity estimated by logistic regression, and the LoD PDF in the domain of the signal intensity. The former is the observed LoD in the domain of the signal intensity for the participant population. Although, we can characterize the LoD in the domain of the signal intensity based on the real-world naked-eye data of the participant population, it is interesting to compare this estimation to one based on easier-to-obtain naked-eye data. Figure 5B shows naked-eye test results, the PPA function, and LoD PDF for trained staff assessing the AT result using a dilution series of recombinant SARS-CoV-2 nucleoprotein (including at a concentration of 0; ie, negatives) with blinded concentration labels. Acknowledging the nonnegligible effect of user heterogeneity [27] and external conditions, the probability functions (cumulative and density) were remarkably similar, as shown in Figures 5A and 5B.

With the analysis shown in Figures 3-5, modeling the input components of the predictive model, we performed our calculations of the PPA function of the qRT-PCR cycles and compared it with the *observed* PPA function (ie, derived from real-world data).

**Figure 6.**
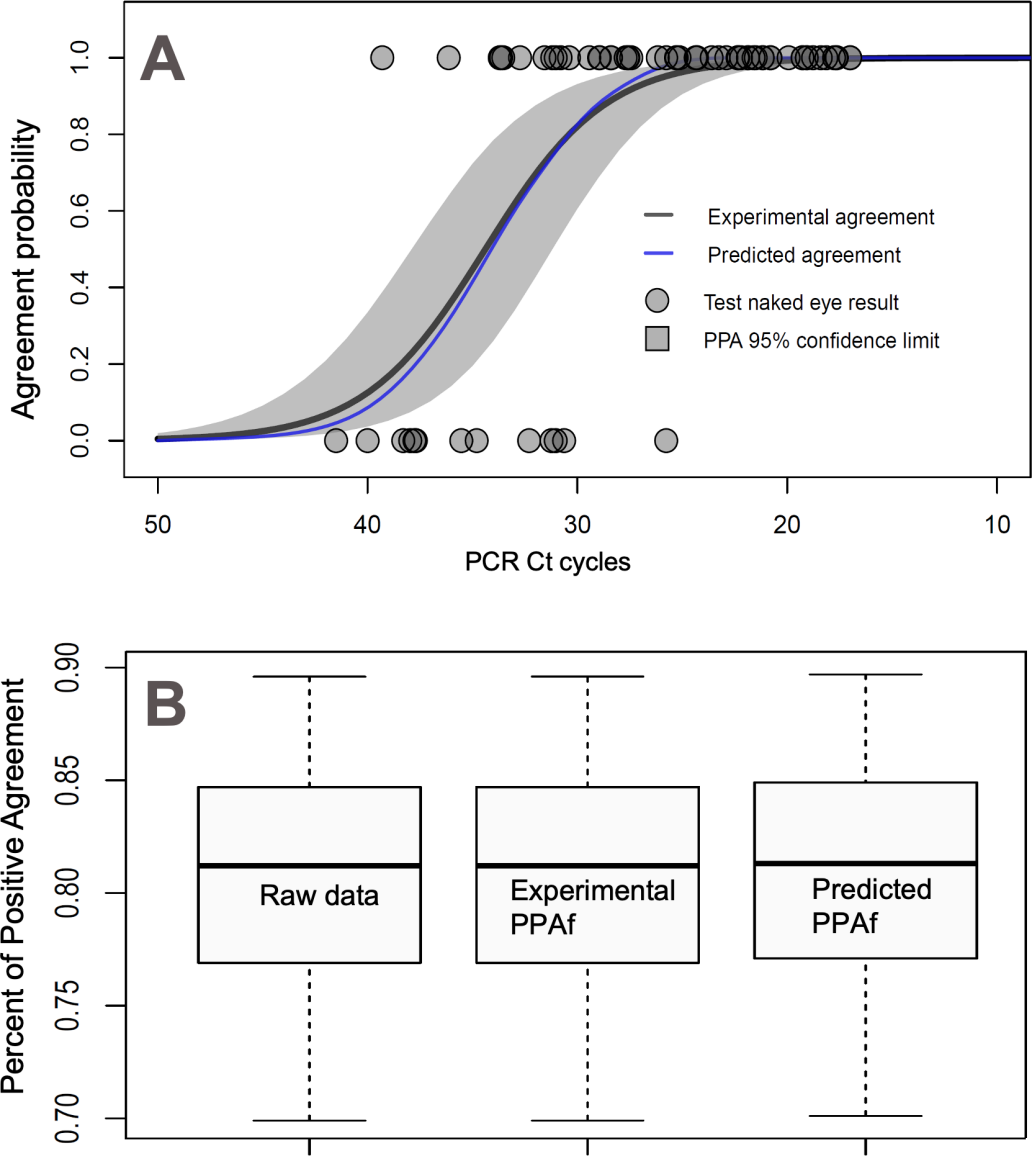
(A) Probability of agreement functions based on experimental real-world data (black curve) and resulting from our predictive model (blue curve). The experimental PPA function was obtained from a logistic regression analysis of the Chelsea project data for the test users; the gray area shows 95% confidence limits on the experimental PPA function. (B) Box plots showing the PPA calculated over the real-world raw data, the observed PPA function, and the predicted PPA function. Box plot bounds are Clopper-Pearson confidence limits for percentiles 50% (box) and 95% (segment). PPA: positive percent agreement; PCR: polymerase chain reaction.

Figure 6A shows the real-world data of binary results obtained with the AT (ie, self-tested and logged data from the Chelsea study) and the corresponding observed PPA as a function of the qRT-PCR cycles. The calculation involved a description of the model uncertainties, illustrated in the plot with confidence intervals. Superimposed, we represented the predicted PPA function in the domain of the qRT-PCR, calculated using our method (Equation 8). The plot clearly shows that the predicted and observed PPA functions are very close and within the figure’s confidence limits. Figure 6B shows a box plot of the overall data PPA for samples collected in the real-world setting for the AT and a comparison of the corresponding predicted real-world PPA according to expression 10. We can verify that both box plots provided close results, within statistical significance.

## Discussion

### Principal Findings

Our predictive framework and the COVID-19 case study indicate that the real-world performance of ATs can be forecast from rapid laboratory measurements together with a statistical characterization of the population’s LoD. First, analysis of test signal intensity and calibration to qRT-PCR can be completed entirely under controlled laboratory conditions—a rapid process that yields precise estimates of baseline AT performance. Second, we show that empirically characterizing the observer population’s LoD distribution for visual interpretation of ATs is feasible.

Across two distinct observer groups and testing settings, the LoD PDFs and the resulting PPA–normalized signal intensity relationships were remarkably similar, suggesting a relatively stable visual decision threshold inherent to this device design. We also demonstrated the practicality of paired AT/qRT-PCR sampling with image uploads, with minimal discrepancy between self-reported results and those recorded by trained staff. Nonetheless, environmental variability (lighting, optics and image compression, and differences among phone cameras), user motivation, and other external factors can broaden the effective LoD distribution.

Because we estimated the PPA as a continuous function of qRT-PCR Ct via logistic regression and then computed the expected sensitivity over a predefined reference Ct distribution, performance can be standardized across sites, time periods, and brands despite heterogeneous sampling. Bayesian fitting used in this study provides a practical path to hierarchical (brand-level) extensions we have recently reported in a complementary publication of the Chelsea clinical study.

Laboratory analyses and our predictive modeling framework provide a fast, standardized path to anticipate clinical performance and guide smarter evaluations of ATs, which is very useful information for the real-world validation of ATs. For regulatory decisions, quantitative summaries should be reported—PPA(Ct) with uncertainty bandwith and expected sensitivity under a preregistered reference Ct distribution—alongside observer LoD distributions (trained vs lay) and robustness to environmental and site effects, all with traceable calibrations and bridging analyses across lots, sites, and readers. After clearance, life cycle quality control should be maintained via routine lot verification, stability tracking, app-enabled field analytics for normalized signal intensity and invalid rates, anomaly detection, and timely label updates when variant-linked changes arise. At minimum, each evaluation should include the PPA(Ct) function with 95% CIs, expected sensitivity under a stated reference Ct distribution, observer LoD PDFs, calibration, and fit diagnostics.

The accurate calibration of the relational components (Figure 2) is fundamental for the fitness of the model. We performed triplicate dilution curves during the process of sample preparation for analysis. Additionally, we verified that thermal inactivation of the virus, as carried out for generating the serial virus dilutions, resulted in marginal loss of capsid protein detection (approximately a 2-fold difference), likely due to heat stability. We also used chemically inactivated virus alongside heat-inactivated virus stocks for signal intensity calibration.

The purpose of this study was to describe a method for the quantification and prediction of AT performance. Further work is underway, as we seek to expand our data for the comparative analysis of performance prediction across several test brands, comparative calibration of the qRT-PCR cycles across service providers, and further characterization of the signal intensity LoD for common ATs.

### Conclusions

An accurate description of the AT signal intensity response conditioned by variables related to viral load, such as concentration of recombinant protein and concentration of inactivated virus, was established under laboratory conditions. These evaluations involved image processing of photographs and human naked-eye assessments using dilution series of the nucleoprotein of the SARS-CoV-2 virus. Modeling the performance on real testing conditions involved integrating the mentioned laboratory evaluation with information on the ability of the observer population to recognize the device’s visual response, which can be described by the LoD PDF of the observer population in the domain of the test signal intensity. We described the overall test performance with the PPA *function* of the qRT-PCR Cts instead of using the common PPA (ie, sensitivity) for segments of clinical data. Our framework predicts PPA versus Ct by linking laboratory-normalized signal intensity–concentration models, observer LoD distributions, and a Ct–viral-load calibration. Conclusions are mathematical and are specific to the disease, assay, and setup. External validity depends on specific Ag-LFTs, user populations (for visual acuity and test interpretation), and qRT-PCR calibration across laboratories. Although the Chelsea dataset supports internal validity, broader clinical evaluation under intended-use conditions is required before generalized clinical claims, particularly for disease targets other than COVID-19. Therefore, the presented methodology has promising applications for the evaluation of ATs, as it involves a quick appraisal of real-world test performance.

## Abbreviations

Ag-LFT: antigen lateral flow test
AT: antigen test
Ct: cycle threshold
IRB: institutional review board
LoD: limit of detection
PCR: polymerase chain reaction
PDF: probability density function
PPA: percent positive agreement
qRT-PCR: quantitative real-time polymerase chain reaction
ROI: region of interest
